# Optical Flow-Based Obstacle Detection for Mid-Air Collision Avoidance

**DOI:** 10.3390/s24103016

**Published:** 2024-05-09

**Authors:** Daniel Vera-Yanez, António Pereira, Nuno Rodrigues, José Pascual Molina, Arturo S. García, Antonio Fernández-Caballero

**Affiliations:** 1 Instituto de Investigación en Informática de Albacete, Universidad de Castilla-La Mancha, 02071 Albacete, Spain; daniel.vera@alu.uclm.es (D.V.-Y.); josepascual.molina@uclm.es (J.P.M.); arturosimon.garcia@uclm.es (A.S.G.); 2 Computer Science and Communications Research Centre, School of Technology and Management, Polytechnic Institute of Leiria, 2411-901 Leiria, Portugal; apereira@ipleiria.pt (A.P.); nunorod@ipleiria.pt (N.R.); 3 Institute of New Technologies—Leiria Office, INOV INESC InovaÇÃO, 2411-901 Leiria, Portugal; 4 Departamento de Sistemas Informáticos, Universidad de Castilla-La Mancha, 02071 Albacete, Spain

**Keywords:** mid-air collision, obstacle detection, computer vision, optical flow, DBSCAN

## Abstract

The sky may seem big enough for two flying vehicles to collide, but the facts show that mid-air collisions still occur occasionally and are a significant concern. Pilots learn manual tactics to avoid collisions, such as see-and-avoid, but these rules have limitations. Automated solutions have reduced collisions, but these technologies are not mandatory in all countries or airspaces, and they are expensive. These problems have prompted researchers to continue the search for low-cost solutions. One attractive solution is to use computer vision to detect obstacles in the air due to its reduced cost and weight. A well-trained deep learning solution is appealing because object detection is fast in most cases, but it relies entirely on the training data set. The algorithm chosen for this study is optical flow. The optical flow vectors can help us to separate the motion caused by camera motion from the motion caused by incoming objects without relying on training data. This paper describes the development of an optical flow-based airborne obstacle detection algorithm to avoid mid-air collisions. The approach uses the visual information from a monocular camera and detects the obstacles using morphological filters, optical flow, focus of expansion, and a data clustering algorithm. The proposal was evaluated using realistic vision data obtained with a self-developed simulator. The simulator provides different environments, trajectories, and altitudes of flying objects. The results showed that the optical flow-based algorithm detected all incoming obstacles along their trajectories in the experiments. The results showed an F-score greater than 75% and a good balance between precision and recall.

## 1. Introduction

Approximately 66 potential and 23 actual mid-air collisions occurred in the United States in 2020 [1]. A total of 75% of actual collisions result in fatalities [1]. As a preventative measure, pilots are instructed to keep one eye on the cockpit, scan the sky for potential threats, and be prepared to maneuver to avoid a potential accident [2,3]. However, this see-and-avoid rule has several important limitations. First, it may be physically impossible for pilots to see approaching aircraft, especially when climbing or descending in an airport traffic pattern. Moreover, the high speed of commercial aircraft makes the see-and-avoid rule inadequate [4]. Pilots are also instructed to follow a pattern by dividing the horizon into regions and taking a moment (1–2 s) to focus before moving on to the next region. Thus, if the horizon is divided into nine regions, the pilot’s eye scans one ninth at a time. In other words, at least 89% of the horizon remains unattended at all times. To make matters worse, the performance of the human eye can be reduced by cloud cover, glare from the sun, fatigue, and many other factors. With the present technologies, which include Secondary Surveillance Radar (SSR) [5], transponders, Traffic Collision Avoidance System (TCAS) [6], and, more recently, Automatic Dependent Surveillance-Broadcast (ADS-B) [7], one might think that mid-air collisions should no longer occur. However, they do happen because these technologies are not mandated equally in all countries, airspaces, or aircraft.

Various safety agencies and pilot associations are encouraging pilots and users of unmanned aerial vehicles (UAVs) to install some form of electronic conspicuity (EC) device on their vehicles to make them more aware of nearby aircraft. An example of such EC technology is Flight Alarm (FLARM, https://flarm.com/, accessed on 6 May 2024). EC devices transmit the position of the host aircraft to other EC devices. The most advanced devices also receive the position of surrounding aircraft and warn the pilot of conflicting traffic [8,9]. FLARM devices also have some limitations. There are incompatibilities, for example, where the communication solution is different due to the use of different frequencies or different protocols [10]. In addition, some devices are active, i.e., they transmit and share their position with others, while others are only passive, i.e., they listen to the transmissions of others but remain invisible to them. Therefore, pilots should rely not only on their eyes to detect threats, but also on an artificial eye that is capable of scanning the sky faster, farther, wider, more sharply, and more consistently [11].

To address the current limitations, the contributions of this work can be summarized as follows:

1. The development of a system leveraging computer vision technology represents a significant advancement in overcoming the limitations inherent in human visual perception. This system operates autonomously, requiring no communication with analogous devices onboard other aircraft to function effectively.
2. The system integrates a suite of sophisticated techniques: light morphological filters, optical flow, the focus of expansion, and Density-Based Spatial Clustering of Applications with Noise (DBSCAN), aimed at averting mid-air collisions. The utilization of traditional computer vision techniques presents a significant advantage over deep neural networks due to the latter’s reliance on extensive training datasets. Such datasets, particularly those concerning mid-air collisions, are exceptionally challenging to acquire, rendering traditional methods more feasible and effective in this context [12].
3. A self-engineered, three-dimensional (3D) simulator, designed to offer a broad spectrum of test environments, is introduced. Within these environments, users have the flexibility to select flight paths over land or sea, adjust the cloud cover, and define aircraft proximity (airprox) scenarios. The simulator is capable of generating datasets from various airprox scenarios, including those with the potential to result in fatal accidents. This functionality enables the researchers to refine the system, enhancing its ability to distinguish between airprox scenarios that are likely to result in a collision and those that are not.

Therefore, the motivation behind this approach is to develop a realistic, optical flow-based collision avoidance system. In such a system, the optical flow of incoming obstacles during flight is calculated in real time using an on-board camera, and the distance and relative speed between the aircraft and the object are estimated. If the system detects a potential collision, it sends a signal to the pilot to take an evasive action, such as changing altitude or direction. The main goal is to make the solution applicable to general aviation. In other words, a sport/light aircraft will detect another aircraft in time to avoid a mid-air collision. The algorithm can also be applied to the UAV field, but there are differences in speed and approximation that can be explored in future work.

The rest of the paper is organized as follows. Section 2 presents the current state of the art in computer-vision obstacle detection. Section 3 describes the proposed solution. Section 4 presents the experimental setup and the evaluation of the algorithm. Finally, Section 5 presents the conclusions of the study.

## 2. Related Work

Since 2005, the interest in utilizing computer vision for aircraft proximity (airprox) and mid-air collision avoidance has significantly increased. Both stereo (two or more sensors) and monocular (single sensor) cameras can perform object detection, but only stereo cameras can calculate the distance from an object with high accuracy [13]. Ref. [14] introduces a collision detection system for Unmanned Aerial Vehicles (UAVs) that leverages stereo vision, utilizing two or more sensors for object detection, as opposed to monocular vision, which relies on a single sensor. The system is capable of processing up to 48 frames per second for images sized 320 × 240, with a power consumption of only 13.5 watts. The choice of stereo cameras is justified by their advantages of being compact, lightweight, and energy-efficient, offering a viable alternative to more power-intensive and bulky methods such as LiDAR or infrared time-of-flight depth sensors.

Monocular camera systems offer distinct advantages over their stereo counterparts, particularly in the context of detecting objects at considerable distances—a critical capability for mid-air collision avoidance systems. Due to their singular lens setup, monocular cameras can streamline the data processing workflow. Leveraging sophisticated algorithms, these systems adeptly extrapolate the trajectory, velocity, and orientation of objects, thus minimizing the need for extensive processing power and computational resources [15]. This efficiency is further bolstered by advanced computer vision techniques including object recognition, motion analysis, and predictive modeling. Such strategies adeptly mitigate the absence of inherent depth perception, employing contextual cues and historical data to accurately gauge potential threats. Moreover, the simplicity of a monocular setup translates to ease in installation and calibration, sidestepping the intricate alignment processes that are essential for stereo cameras to derive precise depth measurements. This comparative simplicity, combined with advanced analytical capabilities, positions monocular cameras as a potent tool in the arsenal against mid-air collisions, balancing technical sophistication with operational pragmatism [16]. These reasons could elucidate why our systematic review [17] determined that monocular cameras are the favored option.

The same review also showed that the most commonly used aircrafts for testing obstacle detection algorithms are multirotor UAVs. In contrast to the cost of testing with real aircraft and helicopters, the increasing availability and affordability of multi-rotor UAVs equipped with on-board cameras and additional computing space has led many researchers to focus on these unmanned vehicles and use them to test their solutions. Prior to real-world testing, most authors begin by testing their solutions in simulators [18]. As UAVs often differ from airplanes and helicopters in speed, weight, and size, the solutions applied to one may not be valid for the other. It is important to review what has been achieved in this regard in the UAV field, to which researchers seem to have paid more attention because of its novelty and affordability.

For example, an obstacle detection technique based on time-to-collision estimates, solved in real time using a model predictive control approach, has been proposed [19]. The algorithm avoided obstacles without being computationally expensive. However, detection failed for images with insufficient features. The authors believed that adding a depth sensor to the system could improve its performance. An algorithm for detecting rapidly approaching obstacles has also been developed [20]. The method detected incoming objects 10 to 40 frames before collision. A Bayesian framework helped identify an object-free region in which the UAV could move to avoid the collision. The solution was tested using videos of drones observing incoming obstacles such as birds, balls, and other drones. A different approach [21] was able to detect an impact between 8 and 10 s in advance, which is close to the recommended 12.5 s reaction time for human pilots. The algorithm uses an image pre-processing approach that uses morphological operations to distinguish potential obstacles, combined with temporal filtering to detect and track persistent features.

In a recent paper, a deep reinforcement learning-based method was presented to enable a quadrotor UAV equipped with a monocular camera to autonomously avoid collisions with obstacles in unstructured and unknown indoor environments [22]. Also, a collision avoidance control method for non-cooperative moving obstacles was introduced for a multicopter with altitude hold mode by using a Lyapunov-like barrier function [23]. The multicopter was able to avoid obstacles as soon as they entered the safety zone and converge to the waypoint. Finally, the autonomous navigation of a UAV in an unknown environment was addressed with a deep reinforcement learning approach [24].

In our approach, we are interested in traditional optical flow-based methods that do not use deep learning [25,26,27]. These techniques have already been applied to flying robots for ego-motion estimation [28], path planning [29], and attitude estimation [30], among other uses. In addition, optical flow shows excellent results in mid-air collision avoidance [29,31,32,33,34]. Optical flow refers to the motion of visual features in an image over time [35,36]. It can be used to estimate the relative motion of objects in a camera’s field of view. Optical flow-based mid-air collision avoidance methods work by analyzing the motion of objects in the camera’s field of view to detect potential collisions. Optical flow-based collision avoidance has been described for multirotor UAVs in urban environments [33]. Recently, a paper described an optical flow-based moving object detection algorithm [34]. The authors of [37] developed an intruder detection system for light and unmanned aerial vehicles. The system uses optical flow and contour block to separate objects from the background. The solution was tested under laboratory conditions using a light aircraft. The results showed that the algorithm can detect the obstacle, but has problems with false positives, especially in good-visibility conditions. Stereovision and optical flow have also been used to avoid collisions between fast moving UAVs [31]. The aforementioned work on 3D path planning for a quadrotor UAV [29] included optical flow-based obstacle avoidance. In addition, a monocular camera, a multirotor UAV, and optical flow were used to avoid incoming obstacles [32].

## 3. Materials and Methods

The algorithm proposed in this paper is an optical flow-based solution that uses a monocular camera to detect incoming flying obstacles. It is an attractive solution due to the relatively low cost, light weight, and reduced computational requirements of the sensors involved. The solution is based on the previously mentioned studies because it uses a monocular camera, morphological operations, and optical flow. However, our algorithm relies entirely on the optical flow vectors to detect incoming obstacles. The algorithm analyzes the direction of the vectors by area to find anomalies that may be caused by an incoming obstacle. The test results presented in Section 4 show promising results in terms of filtering the noise caused by the environment and possible obstacles.

**Figure 1:**
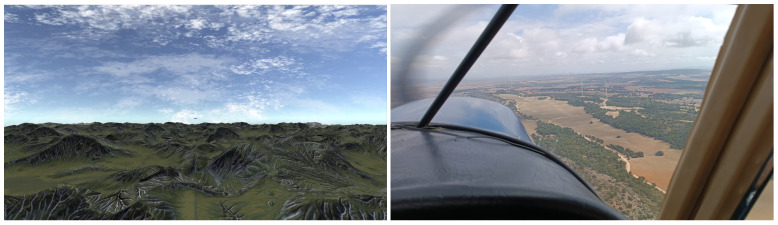
Comparison of simulated and real footage.

In addition, the solution was extensively tested on a realistic mid-air collisions simulator developed by the authors using the game engine Unity (version 2020.3.41f, Unity Technologies, San Francisco, CA, USA). The simulator was used to test the algorithm in different environments and situations, helping us to prove the effectiveness of the detection. A comparison between the simulator and real footage is shown in Figure 1.

**Figure 2:**
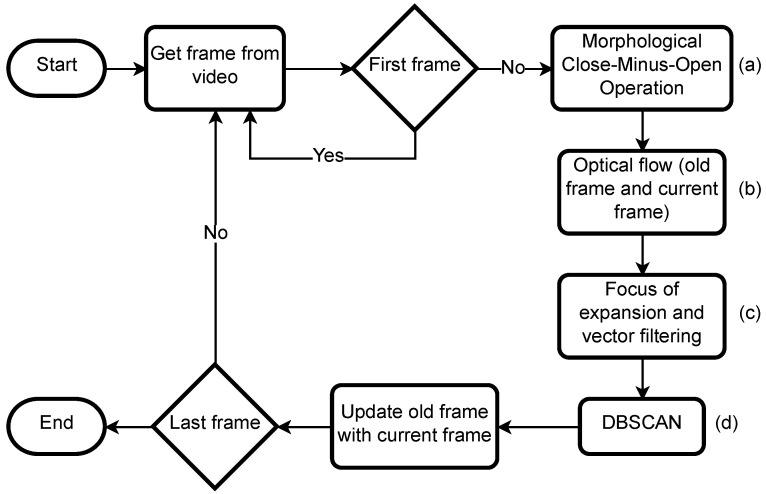
Algorithm flowchart.

The system is engineered to identify obstacles during flight, initiating with imagery captured by a monocular camera. Initially, the close-minus-open (CMO) morphological filter [38] is applied to the image, as depicted in Figure 2a, to diminish noise, segregate elements, and consolidate separated entities. Subsequently, motion vectors are obtained by comparing the current image frame with its predecessor using the Gunnar–Farnebäck (GF) dense optical flow method [39] (see Figure 2b). These optical flow vectors facilitate the determination of the aircraft’s direction through the computation of the focus of expansion (FOE) [40], which is derived from the motion vectors.

In scenarios where the motion vectors of the surrounding environment align with the FOE, an obstacle’s movement will be non-aligned, indicating its presence. The presence of an obstacle is suggested by increased environmental noise, complicating the focus estimation (FE). To compute the FE, the image is partitioned into four equal sections, with the motion vectors in each quadrant being analyzed. These vectors are then cross-referenced across quadrants to pinpoint their intersections, culminating in a compilation of intersection points. To mitigate noise, an averaged intersection point is determined, establishing the FOE. Utilizing the approximated FOE, non-conforming motion vectors are filtered out, as illustrated in Figure 2c. The final step involves clustering the remaining vectors via the DBSCAN algorithm [41,42], with each cluster representing a potential incoming obstacle’s location on Figure 2d.

Previous research has utilized various combinations of the discussed algorithms, yielding promising results, e.g., [40,43,44]. However, our literature review revealed no instances where the close-minus-open (CMO) filter, optical flow, focus of expansion (FOE), and DBSCAN algorithms were employed collectively for the detection of aerial obstacles. Our current methodology does not inherently limit the detection to a single obstacle; however, the primary objective of this study is to evaluate the algorithm’s performance in identifying a singular obstacle. Future work will expand on this foundation, exploring the algorithm’s efficacy in scenarios involving multiple obstacles. The integration of these techniques holds the potential to forge a detection system that is both resilient and precise, capable of operating effectively in varied and challenging conditions. Nonetheless, the system’s ultimate performance and its specific deployment efficacy are contingent upon the quality of the image data and the operational environment’s characteristics.

### 3.1. Morphological Close-Minus-Open Operation

The CMO filter eliminates large regions of clutter, such as large background regions with different mean values, such as sky and mountain regions. It also makes all objects of a given size or smaller and brighter than the background [45]. The CMO algorithm is the difference between the morphological closure and opening of the input scene. The closing operation removes all dark objects or scene regions smaller than a given size. The opening operation removes all light objects or scene regions smaller than a given size [46].

Both closing and opening are combinations of two basic grayscale morphological operations: dilation and erosion. An opening is an erosion followed by a dilation, and a closing is the opposite, a dilation followed by an erosion. The dilation operation grows or thickens objects in a binary image [45]. The grayscale dilation of an input image f(x,y) with a given size k(x,y) is described by Equation (1). The operation involves taking a shifted version of *f*, raising it until it touches *k*, and recording the maximum value of *f* within the support of *k*. This process is repeated for all displacements (x,y) of *f*, and the set of maximum points is the final grayscale dilation result [45].

(1) $$f\left(x,y\right)⊕k\left(x,y\right)=\max_{i,j}\left[f\left(x-i,y-j\right)+k\left(i,j\right)\right]$$

**Figure 3:**
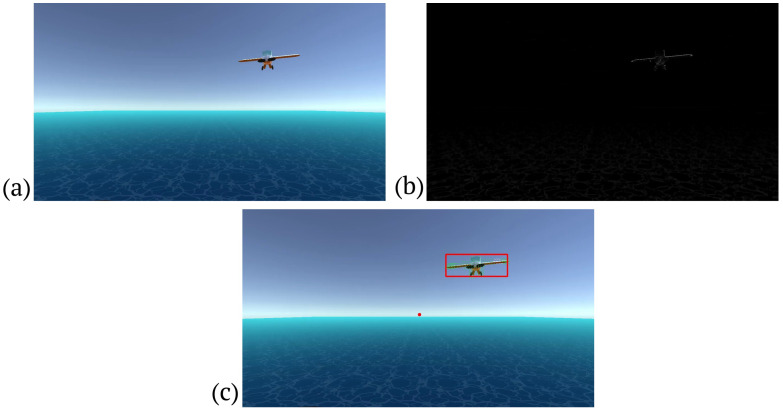
(**a**) Original frame. (**b**) Frame after morphological processing. (**c**) Result of obstacle detection from optical flow; the FOE is represented by a red dot; the red frame indicates the obstacle.

The CMO filter is used in the solution to reduce the noise generated by the clouds, ground, or sun to facilitate the detection of incoming obstacles. Figure 3b shows a frame after applying the CMO filter.

### 3.2. Gunnar–Farnebäck’s Optical Flow

Optical flow is the motion of objects or the camera between every two consecutive frames in a sequence represented by a 2D vector field. Each vector represents the displacement of points from the first frame to the second [47,48,49]. Optical flow can be applied to video stabilization or compression and motion detection [50]. Optical flow assumes that the pixel intensities of an object do not change between frames and that nearby pixels have the same motion. Consider a pixel I(x,y,t) that moves by distance (dx,dy) in the next frame, so that:

(2) I(x,y,t)=I(x+dx,y+dy,t+dt)

Then, the Taylor series approximation of the right-hand side is taken, common terms are removed, and a division by dt is performed to obtain the following equation:

(3) $$f_{x}u+f_{y}v+ft=0$$

where

(4) $$f_{x}=\frac{\delta f}{\delta x};f_{y}=\frac{\delta f}{\delta y};u=\frac{dx}{dt};v=\frac{dy}{dt}$$

$f_{x}$ and $f_{y}$ are image gradients and $f_{t}$ is the gradient over time. (u,v) are unknown. Several ways of solving Equation (4) with two unknown variables are provided and one of them is GF’s optical flow. GF’s algorithm computes the optical flow for all points in the frame. The first step is to approximate each neighborhood of both images through quadratic polynomials. Then, considering these quadratic polynomials, a new signal is constructed via global displacement. Finally, this global displacement is calculated by equating the coefficients in the yields of the quadratic polynomials.

GF’s optical flow provides us with a list of 2D vectors that help us infer the direction in which the plane is moving and detect any incoming obstacles, as shown in Figure 3c.

### 3.3. Focus of Expansion

The intersection of the 3D velocity vector characterizing the camera motion and the projection plane is represented by the FOE in the image plane. Time-to-impact estimation [51] and motion control [28], especially collision warning systems and obstacle avoidance, are prominent applications of FOE. In our implementation, we use the obtained optical flow with GF’s algorithm and compute the estimated FOE. With the resulting FOE, the algorithm can find the velocity vectors that do not coincide with the FOE, indicating the area of a possible incoming obstacle. Figure 3c shows an example of the calculated FOE.

### 3.4. Density-Based Spatial Clustering of Applications with Noise

DBSCAN is a data clustering algorithm that groups data points that are close to each other and marks them as outliers if they are far from any group. It works by starting at a random point in the data and looking for other points that are within a certain distance (eps). If it finds a minimum number of points (min_samples) within that distance, it forms a cluster around those points. It then repeats this process for each point in the cluster until it has gone through all the data [42].

An advantage of DBSCAN is that it can find clusters of any shape, as long as there are enough points within the EPS distance. It is also able to identify points that are outliers, or very different from the rest of the data. DBSCAN is an unsupervised algorithm, which means that it does not require that the data are labeled or that the number of clusters is specified in advance. It is often used in applications where the number and shape of the clusters are unknown, or where the clusters are uneven in size. DBSCAN is used to cluster the vectors that do not match the FOE. The resulting clusters indicate where a possible obstacle is approaching from. Figure 3c shows a cluster of vectors, indicating an approaching obstacle.

## 4. Data and Results

### 4.1. Experimental Setup

We developed a flight simulator using Unity (version 2020.3.41f, Unity Technologies, San Francisco, CA, USA) game engine to test the performance of the algorithm. For this purpose, the simulator rendered the view from the front camera of an aircraft flying across the sky in different environments and cloud covers, while reproducing different airprox scenarios with another aircraft. We selected a generic light aircraft model to simulate potential threats. The specifications of the model match those of Cessna 172 because of its popularity for personal and business travel, as well as flight training. The model accurately represents the dimensions of the real aircraft, with a length of 8.28 m and a wingspan of 11 m. To match the cruising speed of the Cessna 172, the camera-equipped model flew at about 108 knots. During testing, we maintained an altitude of approximately 1000 feet above sea level or ground level (depending on the scenario). We chose this altitude because most mid-air collisions occur below 2000 feet [52].

**Figure 4:**
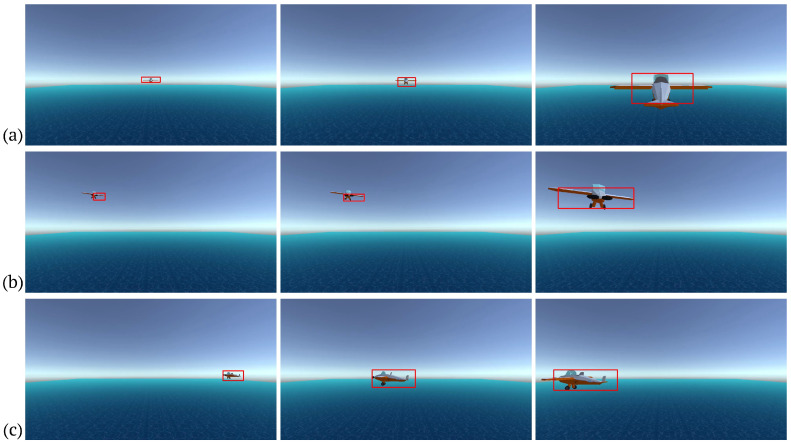
Airprox scenarios. The red frames indicate the detected obstacle. (**a**) Head-on; (**b**) close-in; (**c**) crossing.

For this work, we focused on three of the situations addressed in the internationally agreed-upon rules of the air [3], as follows: head-on approach, convergence, and overtaking. These rules are very important because they describe situations in which a mid-air collision can occur if pilots do not take the appropriate action, i.e., if one gives way to the other. We then simulated three airprox scenarios, one for each of these three rules, focusing on the initial situation described in each one, not on the avoidance actions that should be taken in each case. In this way, we simulated two aircraft approaching head-on, one aircraft crossing the path of the other at 90 degrees, and one aircraft approaching another from behind. We will refer to these scenarios with the usual names: head-on, close-in, and crossing (see Figure 4).

**Figure 5:**
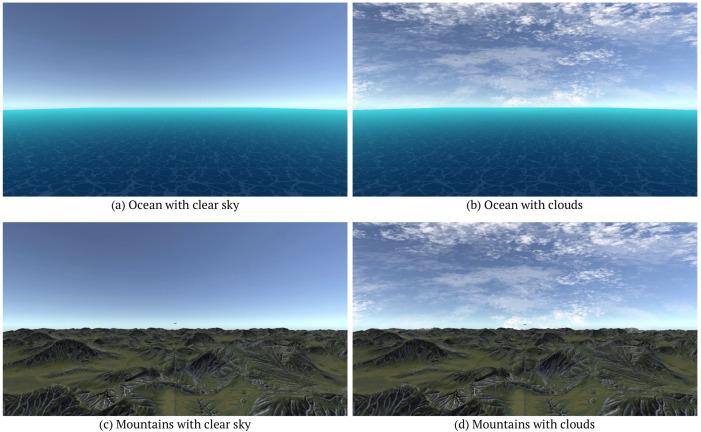
Simulator environments.

The environments selected for testing were flights over mountains and the ocean. In addition, the simulator allowed for clouds to be added to the sky. These environments were chosen to test the algorithm in environments with different noise levels. The resulting surroundings are mountains with clear skies, mountains with clouds, oceans with clear skies, and an ocean with clouds, as shown in Figure 5. Each airprox scenario was tested with three different altitudes for the incoming threat. The obstacle could approach at a higher altitude, a lower altitude, or the same altitude. The difference between the obstacle altitude and the camera for the higher and lower tests was approximately 65 feet. The simulation of four environments, three scenarios, and each individual scenario with a different threat altitude resulted in thirty-six simulations.

The algorithm can detect obstacles as small as 32 pixels wide in an image. Objects smaller than this size are not recognised. The tests are conducted at an altitude of approximately 1000 feet, which is the altitude at which an aircraft approaches an airport or airfield. An “accident”, in the context of this paper, is a collision between two or more flying objects. To be clear, not all approaches result in an accident. The situations that result in accidents are close approaches at the same altitude and all head-on simulations. The other tests simulate very dangerous approaches.

### 4.2. Evaluation

The efficiency of the algorithm was evaluated in terms of detected true positives (TP), true negatives (TN), false positives (FP) and false negatives (FN). In short, TP and TN correspond to correctly classified hits and misses. On the other hand, FP is a false detection (non-obstacle) and FN is a failure to detect a present obstacle. Performance indices such as precision (*P*), recall (*R*), accuracy (Acc), and F-score (*F*) were calculated from the obtained values.

Precision, also known as positive predictive value, tells us the probability of successfully making a correct positive classification. Equation (5) shows the formula for precision.

(5) $$P=\frac{TP}{TP+FP}$$

Furthermore, recall is the sensitivity of the model in identifying a positive class. The mathematical calculation of recall is expressed in Equation (6).

(6) $$R=\frac{TP}{TP+FN}$$

In addition, accuracy represents the number of correctly classified data instances compared to the total number of data instances. All instances are equally important. The accuracy is shown in Equation (7).

(7) $$Acc=\frac{TP+TN}{TP+TN+FP+FN}$$

The F-score takes into account both precision and recall, which are measures of a model’s ability to correctly identify a positive class. This can provide a more complete view of a model’s performance than accuracy alone. The calculation of the F-score is shown in Equation (8).

(8) $$F=2\times \frac{P\times R}{P+R}$$

### 4.3. Results

The overall results of the tests are shown in Table 1. Furthermore, it should be noted that, thanks to the good values of the evaluation variables, it has been possible to observe, in all cases, that the proposal detected the obstacle during a sufficient number of trajectory frames, allowing the obstacle to be avoided. The table shows the precision, recall, accuracy, and F-score for the four environments, the three airprox scenarios, and the three obstacle altitudes. At first glance, looking at the precision, it seems that (1) the ocean environments outperform the mountain environments, (2) the crossing airprox scenario performs better than the other two scenarios, and (3) the obstacle altitude results do not show much variation within each environment/scenario block.

In fact, after calculating the averages for each block, it can be confirmed that obstacle altitude leads to small differences, as shown in Table 2.

Since obstacle altitude does not play a role in this study, Table 3 provides a more compact view of the results.

#### 4.3.1. Results According to Environment

Table 1 showed that the two ocean environments outperformed the mountain environments in terms of precision. This is confirmed in Table 4, which reports the results by grouping the data from the two ocean and the two mountain environments. The opposite is true for recall, which causes the accuracy and F-score to be quite similar.

The better precision of the ocean compared to mountainous environments is due to the fact that a calm ocean has fewer irregularities, which translates into less noise and fewer false positives. An interesting future test would be to check the algorithm’s performance on a rough ocean. The effect of noise on the algorithm can be seen by comparing the accuracy of cloudy scenarios compared to clear sky scenarios (see Table 5).

As you can see, there is one outstanding scenario in terms of precision. The ocean with clear skies has an accuracy of 91.98%; this is because the clouds produce irregularities in the image (noise), and the higher the noise, the higher the possibility of false positives.

In the future, the noise caused by environmental irregularities such as clouds or mountains could be reduced by analyzing whether there are significant color variations in the obtained clusters. A color variation in a cluster compared to its neighbors could mean that an obstacle has been encountered. Alternatively, it could be due to a variation in the environment, which could mean that the cluster should be ignored.

#### 4.3.2. Results According to Airprox Scenario

In addition, Table 1 showed that the crossing airprox scenario outperformed both the close-in and the head-on scenarios in terms of precision. This is now confirmed in Table 6, but not so much for precision as for recall, accuracy, and F-score.

This is obviously due to the 100% recall obtained for the crossing airprox scenario. This is because the optical flow of the moving obstacle is in a completely different direction to the optical flow of the camera movement. Therefore, the algorithm detects the threat more easily. This is comparable to the worst recall score (see 37.10% for ocean with clouds, head-on, and same altitude in Table 1). In this case, the optical flow of the threat is aligned with the optical flow of the camera feed. This makes it more difficult for the algorithm to separate the obstacle from the background, which explains the lower recall. We believe that it would be possible to improve recall in such cases by using the motion vectors to estimate the speed of the aircraft [53].

### 4.4. Comparison

One goal of this study is to provide pilots with a user interface that can detect potential obstacles in time to prevent fatal accidents. With an accuracy of over 75%, the solution shows a good performance regardless of weather conditions. It is important to note that every obstacle was detected in all tests. This is a step forward in achieving the proposed goal. When comparing our algorithm with other, similar studies, it should be noted that a fair comparison is not always easy. Most approaches to mid-air collision avoidance do not provide the efficiency parameters traditionally used in computer vision. Nevertheless, we were able to find some works for comparison to improve our future work.

Reference [54] outlines a novel vision-based sense-and-avoid (SAA) algorithm tailored to Unmanned Aerial Vehicles (UAVs), aimed at preventing mid-air collisions through the detection and tracking of approaching aircraft. The algorithm processes video data from an onboard camera, employing these techniques to accurately identify and track potential collision threats. Key operations include the sampling of new particles around detected objects, likelihood estimation for particle weighting based on proximity to objects, and the selection of high-likelihood particles to represent objects posing a collision risk. The effectiveness of tracking is enhanced by continuously adjusting the particle set through resampling, ensuring that computational resources are focused on the most probable object paths.

To validate the algorithm, the authors of [54] conducted flight experiments involving two UAVs equipped with navigation systems, where one UAV also carried an onboard camera for implementing the SAA tasks. These UAVs flew along a predefined circular path in opposite directions, simulating potential collision scenarios. The experiments demonstrated the algorithm’s ability to detect approaching aircraft at a practical distance, enabling the execution of avoidance maneuvers. The successful detection and tracking of the UAV without false alarms in a real flight situation underscore the algorithm’s potential for enhancing the safety and autonomy of UAV operations.

Although the authors of the referenced study did not release the dataset used to validate their solution, they provided comprehensive documentation of their algorithm. This detailed documentation enabled us to replicate their approach and apply the algorithm within our simulator for testing purposes. We evaluated their solution using identical tests and parameters to those used in the assessment of our algorithm, ensuring a consistent and fair comparison. Table 7 displays the results, categorized by airprox scenario and environment, while additional comparisons are detailed in Appendix A.

Table A3 illustrates that algorithm [54] achieves higher precision in mountainous environments than in oceanic ones. This disparity arises from the algorithm’s reliance on below-horizon features to identify obstacles. Despite the ocean tests yielding better overall results for our solution, it was during the mountain teststhat algorithm [54] demonstrated superior precision. This outcome can be attributed to our solution’s heightened sensitivity to noise relative to the proposed algorithm, which necessitates a rich feature set for effective obstacle detection.

In the context of airprox scenarios (Table A5), both solutions delivered comparable outcomes, with no particular scenario showing marked superiority. However, when dissecting the results further, our solution exhibited a notable performance in crossing scenarios, attributed to its enhanced sensitivity to abrupt movements. This contrast did not extend to altitude tests (Table A2), where both algorithms performed similarly, underscoring a general parity in their abilities.

Despite each algorithm excelling under different test conditions, their overall performance was commendably robust. In the future, the aim is to integrate the strengths of both algorithms to forge a more reliable solution, hoping to achieve promising advancements in obstacle detection efficacy.

We also conducted a comparative analysis of our solution and two other studies [55,56], basing the comparison on the results reported by the authors of these works. The first study uses the CMO morphological filter as we did, to reduce noise and highlight small features [55]. The authors decided to compare two algorithms for obstacle detection: the Hidden Markov Model (HMM) and Viterbi-based target detection. Both algorithms were preceded by the CMO filter. The authors used two fixed-wing UAVs to collect the test data. The tests included head-on and intersection scenarios. They also collected test data using a Cessna 172 aircraft. The results showed that their solution could detect obstacles from 400 to about 900 m. At these distances, the system could provide 8 to 10 s of warning. The tests also focused on the effect of jitter on detection. The authors found that the HMM filter was more sensitive to the effects of jitter. They suggested that the effects of jitter could be reduced by using gyroscopes, accelerometers, and other inertial sensors, or by directly tracking salient features. The effect of jitter was not considered in our tests and would be a valuable topic for future work.

The second study uses a very different approach [56]. It uses a stereo camera with an independent rotational degree of freedom to actively sense the obstacles. The detection starts with a combination of the Kalman filter and the SORT algorithm to define regions of interest. Then, the actual obstacle is detected using an algorithm that uses the YOLOv3 deep convolutional neural network.

As mentioned at the beginning of this paper, we based our solution on a traditional optical flow algorithm. However, the door is open to compare traditional methods with modern optical flow-based methods using deep learning, such as Full Flow [57], FlowNet 2.0 [58], LiteFlowNet [59], and 3D-FlowNet [60], among others.

## 5. Conclusions

In this research, we investigated the development of an optical flow-based airborne obstacle detection algorithm to avoid mid-air collisions. The goal was to develop an application to alert a pilot of incoming obstacles in real time to prevent mid-air collisions. The proposal used the CMO filter to reduce the noise in the environment. Then, Gunnar–Farnebäck’s optical flow algorithm is applied to obtain the velocity vectors. The vectors are filtered and grouped using the focus of expansion and the data clustering algorithm called DBSCAN. The resulting cluster can indicate a possible incoming obstacle.

We evaluated the algorithm using an in-house simulator. The dataset of images used in the tests was obtained using the simulator. The tests showed that although the intruder was identified in all tests, the performance was affected by the motion of the incoming obstacle, which caused a significant amount of false positives. Consistent with the limitations of optical flow, when the flying object matches the motion of the environment, it is harder to detect. However, the results of the simulations showed that the system can identify incoming obstacles under normal weather conditions. In the future, the authors plan to explore ways to mitigate the matching motion, such as checking the color range of the detected clusters and adding deep neural networks.

In future work, we will evaluate the presented algorithm using real-time tests to see if it is fast enough to warn a pilot of an approaching obstacle. A comparison with the human eye will tell us if our proposal can outperform human classification. The authors also want to investigate the most efficient and least invasive way to warn a pilot of an approaching obstacle. 

## Figures and Tables

**Table 1:** Simulation results.

Environment	Airprox Scenario	Obstacle Altitude	Precision	Recall	Accuracy	F-Score
ocean with clouds	close-in	higher	75.00%	55.85%	61.44%	64.02%
		same	77.39%	46.11%	56.95%	57.79%
		lower	80.24%	69.43%	70.23%	74.44%
	head-on	higher	77.78%	45.90%	80.38%	57.73%
		same	75.41%	37.10%	60.26%	49.73%
		lower	72.00%	48.65%	73.47%	58.06%
	crossing	higher	81.88%	100.00%	88.69%	90.04%
		same	86.15%	100.00%	91.55%	92.56%
		lower	81.29%	100.00%	88.29%	89.68%
ocean with clear sky	close-in	higher	96.80%	58.45%	71.79%	72.89%
		same	94.57%	42.44%	61.32%	58.59%
		lower	95.56%	63.86%	75.00%	76.56%
	head-on	higher	89.66%	47.27%	89.26%	61.90%
		same	88.64%	42.39%	79.21%	57.35%
		lower	77.50%	43.06%	82.27%	55.36%
	crossing	higher	94.92%	100.00%	97.35%	97.39%
		same	95.69%	100.00%	97.77%	97.80%
		lower	94.69%	100.00%	97.29%	97.27%
mountains with clear sky	close-in	higher	60.33%	82.22%	63.40%	69.59%
		same	60.13%	82.88%	67.48%	69.70%
		lower	65.87%	85.94%	70.12%	74.58%
	head-on	higher	61.40%	92.11%	81.75%	73.68%
		same	60.94%	95.12%	79.85%	74.29%
		lower	65.12%	93.33%	85.71%	76.71%
	crossing	higher	68.94%	100.00%	75.85%	81.62%
		same	71.52%	100.00%	77.94%	83.39%
		lower	67.66%	100.00%	74.65%	80.71%
mountains with clouds	close-in	higher	62.30%	72.15%	59.35%	66.86%
		same	65.67%	71.54%	66.25%	68.48%
		lower	65.05%	81.76%	66.42%	72.46%
	head-on	higher	67.65%	79.31%	90.17%	73.02%
		same	67.27%	80.43%	83.93%	73.27%
		lower	73.91%	80.95%	87.73%	77.27%
	crossing	higher	71.33%	100.00%	77.47%	83.27%
		same	72.55%	100.00%	78.13%	84.09%
		lower	71.15%	100.00%	76.92%	83.15%

**Table 2:** Results according to obstacle altitude.

Obstacle Altitude	Precision	Recall	Accuracy	F-Score
higher	74.98%	74.29%	76.86%	74.63%
same	76.19%	67.06%	73.18%	71.33%
lower	76.81%	78.20%	78.26%	77.50%

**Table 3:** Results according to airprox scenario and environment.

Environment	Airprox Scenario	Precision	Recall	Accuracy	F-Score
ocean with clouds	close-in	77.73%	57.14%	62.92%	65.86%
	head-on	74.83%	42.47%	70.89%	54.19%
	crossing	83.05%	100.00%	89.48%	90.74%
ocean with clear sky	close-in	95.94%	54.89%	69.36%	69.77%
	head-on	83.88%	60.79%	73.96%	70.49%
	crossing	95.10%	100.00%	97.47%	97.49%
mountains with clear sky	close-in	62.10%	83.69%	66.93%	71.30%
	head-on	62.20%	93.58%	82.31%	74.73%
	crossing	69.34%	100.00%	76.12%	81.90%
mountains with clouds	close-in	64.21%	75.29%	63.89%	69.31%
	head-on	69.63%	80.34%	87.30%	74.60%
	crossing	71.68%	100.00%	77.50%	83.51%

**Table 4:** Results according to environment.

Environment	Precision	Recall	Accuracy	F-Score
ocean	85.81%	67.02%	76.01%	75.26%
mountains	66.53%	88.34%	73.96%	75.90%
Total	75.55%	75.57%	75.11%	75.56%

**Table 5:** Results according to environment and cloud cover.

Environment	Precision	Recall	Accuracy	F-Score
ocean with clouds	79.51%	66.27%	73.10%	72.29%
ocean with clear sky	91.98%	67.67%	78.65%	77.97%
mountains with clear sky	65.16%	91.71%	73.54%	76.19%
mountains with clouds	67.98%	85.17%	74.37%	75.61%

**Table 6:** Results by airprox scenario.

Airprox Scenario	Precision	Recall	Accuracy	F-Score
Close-in	73.05%	65.34%	65.83%	68.98%
Head-on	74.72%	62.04%	77.39%	67.79%
Crossing	78.55%	100.00%	85.60%	87.98%

**Table 7:** Results obtained by the airprox scenario and the environment of the solution presented by [54].

Environment	Airprox Scenario	Precision	Recall	Accuracy	F-Score
ocean with clouds	close-in	67.34%	67.64%	63.20%	67.49%
	head-on	75.96%	55.82%	70.04%	64.35%
	crossing	74.39%	77.07%	68.26%	75.70%
ocean with clear sky	close-in	83.03%	75.29%	74.63%	78.97%
	head-on	67.89%	58.11%	75.52%	62.62%
	crossing	74.33%	76.94%	68.77%	75.62%
mountains with clear sky	close-in	82.77%	90.69%	84.33%	86.55%
	head-on	82.42%	83.95%	89.70%	83.18%
	crossing	86.03%	98.85%	90.83%	92.00%
mountains with clouds	close-in	96.03%	98.37%	97.03%	97.19%
	head-on	79.84%	79.80%	79.63%	79.82%
	crossing	74.33%	76.94%	68.77%	75.62%

## Data Availability

The data presented in this study are available on request from the corresponding author.

## Abbreviations

The following abbreviations are used in this manuscript:

ADS-B	Automatic Dependent Surveillance Broadcast
CMO	Close-Minus-Open
EC	Electronic Conspicuity
FLARM	FLight alARM
FOE	Focus of expansion
GF	Gunnar–Farnebäck
HMM	Hidden Markov Model
ROS	Robot Operating System
SSR	Secondary Surveillance Radar
TCAS	Traffic Collision Avoidance System
UAV	Unmanned aerial vehicle

## Appendix A

This appendix presents the results obtained after testing the solution presented in [54].

**Table A1:** Ref. [54] simulation results.

Environment	Airprox Scenario	Obstacle Altitude	Precision	Recall	Accuracy	F-Score
ocean with clouds	close-in	higher	62.84%	65.03%	58.82%	63.92%
		same	70.86%	68.59%	66.06%	69.71%
		lower	68.24%	69.18%	64.48%	68.71%
	head-on	higher	72.58%	60.00%	72.51%	65.69%
		same	75.86%	57.89%	70.89%	65.67%
		lower	79.37%	51.02%	67.03%	62.11%
	crossing	higher	77.56%	80.13%	71.98%	78.83%
		same	74.84%	75.80%	67.90%	75.32%
		lower	71.10%	75.46%	65.25%	73.21%
ocean with clear sky	close-in	higher	82.76%	73.17%	71.25%	77.67%
		same	84.55%	72.66%	75.93%	78.15%
		lower	82.09%	80.29%	77.03%	81.18%
	head-on	higher	75.00%	49.45%	74.48%	59.60%
		same	64.52%	57.97%	73.85%	61.07%
		lower	64.71%	70.97%	78.46%	67.69%
	crossing	higher	75.00%	78.95%	69.86%	76.92%
		same	69.93%	71.81%	63.33%	70.86%
		lower	79.17%	81.20%	74.59%	80.17%
mountains with clear sky	close-in	higher	82.39%	92.13%	85.11%	86.99%
		same	81.40%	92.92%	85.45%	86.78%
		lower	84.09%	88.10%	82.80%	86.05%
	head-on	higher	82.61%	92.68%	94.18%	87.36%
		same	83.33%	91.67%	90.36%	87.30%
		lower	81.13%	70.49%	84.36%	75.44%
	crossing	higher	83.94%	99.14%	89.59%	90.91%
		same	88.98%	99.12%	92.89%	93.78%
		lower	85.40%	98.32%	90.09%	91.41%
mountains with clouds	close-in	higher	96.85%	99.19%	97.88%	98.01%
		same	95.58%	98.18%	96.86%	96.86%
		lower	95.65%	97.78%	96.39%	96.70%
	head-on	higher	95.92%	87.04%	96.96%	91.26%
		same	91.18%	92.54%	95.69%	91.85%
		lower	91.76%	96.30%	96.54%	93.98%
	crossing	higher	72.56%	99.17%	83.75%	83.80%
		same	73.51%	98.23%	84.39%	84.09%
		lower	72.22%	99.15%	83.21%	83.57%

**Table A2:** Ref. [54] results according to obstacle altitude.

Obstacle Altitude	Precision	Recall	Accuracy	F-Score
higher	79.26%	81.40%	81.59%	80.32%
same	79.55%	81.82%	81.33%	80.67%
lower	80.61%	83.33%	82.03%	81.95%

**Table A3:** Ref. [54] results according to environment.

Environment	Precision	Recall	Accuracy	F-Score
ocean	74.03%	70.52%	69.80%	72.34%
mountains	83.60%	88.42%	85.11%	86.09%
Total	78.85%	79.07%	77.42%	84.09%

**Table A4:** Ref. [54] results according to environment and cloud cover.

Environment	Precision	Recall	Accuracy	F-Score
ocean with clouds	71.82%	68.93%	66.75%	70.35%
ocean clear sky	76.51%	72.29%	73.00%	74.34%
mountains clear sky	84.01%	92.60%	88.03%	88.10%
mountains with clouds	83.24%	84.96%	82.33%	84.09%

**Table A5:** Ref. [54] results according to airprox scenario.

Airprox Scenario	Precision	Recall	Accuracy	F-Score
Close-in	81.70%	82.19%	79.44%	81.94%
Head-on	76.93%	69.41%	78.72%	72.97%
Crossing	77.08%	81.71%	74.06%	79.33%
